# When is rPPG physiologically valid for stress and workload monitoring? A PRISMA-ScR scoping review

**DOI:** 10.3389/fdgth.2026.1855425

**Published:** 2026-08-19

**Authors:** Arshad Nasser, Malak Baslyman, Sami Elferik

**Affiliations:** King Fahd University of Petroleum & Minerals, Dhahran, Saudi Arabia

**Keywords:** contactless sensing, heart rate variability, imaging PPG, mental workload, pulse rate variability, remote photoplethysmography, rPPG, stress detection

## Abstract

**Introduction:**

Remote photoplethysmography (rPPG) enables non-contact estimation of cardiovascular signals from ordinary cameras and has been increasingly applied to stress and mental workload assessment using heart rate (HR), heart rate variability (HRV), and pulse rate variability (PRV). However, reported performance varies substantially across studies, and stress-classification results are often presented without sufficient physiological validation, limiting interpretability and generalizability.

**Methods:**

This paper presents a PRISMA-ScR-guided scoping review of rPPG-based stress and mental workload research published between January 2010 and March 2026. Searches across Scopus, IEEE Xplore, PubMed, ACM Digital Library, and Google Scholar identified 20 empirical studies meeting predefined inclusion criteria (stress, mental workload, anxiety, and stress-related arousal outcomes); additional references were retained for background (methods, datasets/benchmarks, bias, and wearable comparisons) and were excluded from PRISMA counts. The literature is synthesized across laboratory, academic, online-interaction, and telehealth contexts, with emphasis on signal-processing pipelines, physiological targets, validation strategies, and deployment conditions.

**Results:**

The reviewed evidence indicates that rPPG supports robust non-contact heart-rate estimation, while HRV/PRV reliability degrades under head motion, illumination variability, and short analysis windows commonly used for stress inference. Many studies rely on protocol labels or wearable references without agreement against electrocardiography or high-quality contact photoplethysmography, constraining physiological validity.

**Discussion:**

Based on these findings, this review provides a validity-oriented synthesis and reporting recommendations to support reliable rPPG-based stress and workload assessment in ecologically relevant settings.

## 1 Introduction

Psychological stress and mental workload play critical roles in cognitive performance, emotional well-being, and long-term health. Elevated stress levels affect attention, decision-making, and physiological regulation, making continuous monitoring increasingly important in domains such as telehealth, education, transportation, and human–computer interaction. While a broad literature exists on remote photoplethysmography (rPPG) for vital-sign estimation, this review focuses specifically on empirical studies that apply rPPG to infer stress, mental workload, arousal, or closely related mental-state constructs, and that report quantitative experimental results. Many stress-assessment systems rely on cardiovascular markers such as heart rate (HR) and heart rate variability (HRV), which reflect autonomic nervous system (ANS) activity and provide sensitive indicators of cognitive and emotional states.

Traditional physiological sensing methods, including electrocardiography (ECG) and contact photoplethysmography (PPG), offer accurate HR and HRV measurements but require skin contact. Even wrist-worn wearable devices suffer from motion artifacts, variable contact pressure, and poor peripheral perfusion, which degrade HRV reliability in real-world environments (1, 2). This aligns with broader guidance on wearable PPG limitations and best practices for cardiovascular monitoring (3). Recent evaluations confirm that wrist-based PPG often underperforms compared to ECG for acute stress detection, whereas alternative form factors such as earbud PPG show improved performance (4). These limitations highlight a growing need for unobtrusive, non-contact sensing methods capable of monitoring stress outside controlled laboratory settings.

Remote photoplethysmography (rPPG) offers a promising alternative by enabling the recovery of pulsatile blood-volume changes from standard cameras. Building on foundational work in imaging photoplethysmography and blind source separation (5–7), subsequent advancements such as CHROM (8), POS (9), and deep learning–based models like DeepPhys (10) have significantly improved signal extraction under motion and illumination changes. Broad overviews of rPPG methods, performance, and measurement challenges are available in recent surveys and reviews (11–13). These developments have established rPPG as a viable method for non-contact estimation of HR, HRV, and pulse rate variability (PRV) across a range of environments.

Among the empirical rPPG studies that explicitly target stress, mental workload, or anxiety as primary outcomes, a limited but growing body of work has investigated the feasibility of deriving cardiovascular markers from facial video under controlled and semi-naturalistic conditions. In the last decade, researchers have increasingly applied rPPG to stress-related arousal cues. Early work demonstrated that webcam-based rPPG can recover HRV/PRV changes under controlled mental stress paradigms and support stress vs. baseline discrimination (14–16). More recent studies have shown robust performance in academic settings (17), online meetings (18), livestream telehealth scenarios (19), and controlled mental arithmetic or social evaluative stressors (20–22). Recent work has also explored camera-based peripheral hemodynamics for quantifying stress-induced sympathetic activity under controlled perturbations (23). However, rPPG performance can degrade substantially in uncontrolled “in-the-wild” webcam conditions, underscoring the importance of ecologically valid evaluation (24). Several PRISMA-included deep-learning studies report high within-dataset accuracy for stress classification on public datasets (25–30); however, without subject-independent evaluation, artifact controls (e.g., motion/illumination/compression), and reference-anchored validation of HRV/PRV, such results may reflect condition-correlated confounds rather than autonomic stress responses. Multimodal approaches combining rPPG with thermal or behavioral signals further enhance sensitivity to subtle autonomic changes and affective arousal (including arousal/valence) (31–33).

Despite this progress, the literature remains fragmented. Studies differ widely in stress-induction protocols, camera modalities, preprocessing methods, HRV feature sets, and ground-truth measurements. Findings are inconsistent regarding the reliability of rPPG for HRV estimation. Some report close agreement with contact PPG and ECG (34, 35), while others note reduced accuracy under motion, low light, or naturalistic conditions (36, 37). The growth of wearable PPG–based stress assessment adds further complexity, with mixed evidence on whether wearable HRV provides a suitable benchmark for evaluating rPPG systems (38). These methodological inconsistencies limit comparability across studies and make it difficult to evaluate rPPG’s true potential for practical stress monitoring.

### 1.1 Novelty relative to existing rPPG surveys

Several recent surveys comprehensively review rPPG algorithms and performance for vital-sign estimation, with emphasis on heart rate (and in some cases ${\mathrm{SpO}}_{2}$) under diverse capture conditions (11–13). In contrast, the present work focuses on validity-oriented synthesis of empirical rPPG stress/workload studies: the evidential conditions under which rPPG-derived HRV/PRV can support claims about autonomic stress/workload responses.

Our goal is therefore not to re-survey rPPG extraction methods broadly, but to synthesize the stress/workload literature through a validity-oriented lens (ground truth strength, window length, reference modality, signal quality handling, and evaluation protocol), highlighting when HRV/PRV-based inference is defensible and when “do-not-infer” behavior is warranted. Table 1 positions this review relative to widely cited rPPG surveys and highlights the validity-oriented focus on HRV/PRV-based stress/workload inference.

**Table 1:** Positioning of this review relative to widely cited rPPG surveys.

Survey/Review	HR/${\mathrm{SpO}}_{2}$ focus	Stress/workload focus	HRV/PRV validity rules	Primary contribution
Chen et al. (IEEE TIM) (11)	✓	–	Limited	Methods landscape and HR measurement challenges.
Xiao et al. (12)	✓	–	Limited	HR measurement review across algorithms and settings.
Pirzada et al. (IEEE Sensors J.) (13)	✓	–	Limited	HR/${\mathrm{SpO}}_{2}$ review and measurement considerations.
This review	✓(as prerequisite)	✓	✓	Validity ladder + scenario matrix + reporting guidance for defensible stress/workload inference.

#### 1.1.1 Scope and constructs

In this review, we treat *stress* and *mental workload* as related but distinct constructs. Stress refers to affective and physiological responses to perceived demands or threat, while mental workload refers to cognitive resource demands during task performance. Because many rPPG papers use overlapping proxies (e.g., HRV/PRV features) and label sources (protocol blocks, self-report), we group stress, workload, and closely related arousal/anxiety outcomes under an “ANS-activation” umbrella for mapping, but we report label types and ground-truth strength explicitly in the synthesis. Emotion and valence-focused rPPG studies are discussed only where they provide methodological context or highlight confounds relevant to stress and workload inference, and are not counted as PRISMA-included studies.

To address these gaps, this review provides a structured synthesis of rPPG research applied specifically to stress, arousal, and mental workload assessment. The contributions of this work are fourfold:

- We analyze the evolution of rPPG signal processing, from classical methods to modern deep learning, and evaluate their implications for stress-related physiological measurements.
- We consolidate empirical evidence on the use of rPPG for detecting stress, anxiety, and cognitive workload across diverse experimental paradigms.
- We critically compare rPPG performance with contact PPG, wearable devices, and ECG, clarifying when rPPG is viable for HRV-based stress assessment.
- We identify key challenges such as motion artifacts, illumination changes, HRV reliability, dataset limitations and outline a roadmap for future research, including multimodal fusion and deployment in real-world and pediatric environments.

By integrating algorithmic, physiological, and application-level perspectives, this review aims to clarify the current capabilities and limitations of rPPG for mental state assessment and to provide a foundation for the next generation of non-contact stress monitoring systems.

## 2 Background: PPG, rPPG, and stress physiology

### 2.1 Photoplethysmography (PPG)

Photoplethysmography (PPG) is an optical sensing technique that measures blood volume changes in peripheral microvascular tissue using light absorption. A light source illuminates the skin, and a photodetector captures the variations in reflected or transmitted light corresponding to pulsatile blood flow. Contact PPG sensors such as finger clips, ear-clip devices, and wrist-worn wearables provide reliable heart rate (HR) and, under ideal conditions, heart rate variability (HRV). However, contact PPG performance is sensitive to motion artifacts, contact pressure, and sensor placement, particularly at the wrist where perfusion is variable (1, 2). These limitations often degrade HRV accuracy during stress tasks or non-resting states (4, 38).

### 2.2 Remote photoplethysmography (rPPG)

Remote photoplethysmography (rPPG) extends PPG into a non-contact domain by estimating pulsatile blood-volume changes from camera recordings. Initially demonstrated using ambient light and simple video-based optical measurements (5), rPPG gained momentum with the introduction of blind source separation and independent component analysis for pulse extraction (6, 7). Modern rPPG pipelines typically involve four stages: (1) face detection and region-of-interest (ROI) selection, (2) spatial averaging of pixel intensities, (3) projection into physiologically informative color subspaces, and (4) temporal filtering for pulse-wave recovery. ROI definition (including optimal sub-regions and skin-tissue masking) is a key determinant of robustness and has been studied explicitly in recent work (39, 40).

Two widely used chrominance-based algorithms; CHROM (8) and POS (9) project RGB signals into optimized color combinations that enhance the pulsatile component while suppressing motion and illumination variations. These methods remain the foundation for much of today’s signal-processing research. More recently, deep learning models such as DeepPhys (10) and multi-task spatiotemporal networks have improved robustness under challenging real-world lighting and movement conditions. Despite these advances, rPPG signals remain sensitive to facial motion, occlusions, skin reflectance properties, and camera quality, which directly affect the reliability of HRV estimation (36, 37).

### 2.3 Stress and mental workload physiology

Stress and mental workload trigger physiological changes mediated by the autonomic nervous system (ANS). Cognitive and emotional stressors typically elicit sympathetic activation and concurrent vagal (parasympathetic) withdrawal. These are parallel regulatory responses of the ANS, not a sequential chain. Sympathetic activation does not cause parasympathetic withdrawal; both branches respond contemporaneously to the central stress signal (41). This dual-branch response increases heart rate and reduces overall beat-to-beat variability. However, HRV responses are not uniform across stressors, individuals, or metrics. Time-domain measures such as RMSSD and its frequency-domain correlate HF-HRV primarily reflect parasympathetic (vagal) modulation, and are the most widely used stress-sensitive indices. Meta-analytic evidence confirms that HF-HRV and RMSSD specifically decrease under psychological stress, while HR elevates non-specifically under both physical and psychological load (42). SDNN captures combined sympathetic and parasympathetic variance and is more sensitive to longer recording windows. The LF/HF ratio is frequently interpreted as a sympathovagal balance index, but its physiological meaning is contested: respiratory artefacts, recording duration, and stationarity all affect spectral estimates, making LF/HF particularly unreliable in short analysis windows (43). For rPPG-based stress systems, time-domain metrics (RMSSD, SDNN) are therefore more appropriate than LF/HF for short-window inference. Spectral estimates derived from ultra-short rPPG windows should be interpreted with caution (36, 44).

PPG and rPPG can estimate pulse rate variability (PRV), which correlates with HRV but is more susceptible to noise and measurement artifacts. Studies show that rPPG-derived HRV can capture stress-induced autonomic changes under controlled conditions (14–16, 20), while PRV from contact or remote PPG often underestimates variability during motion or spontaneous facial behavior (34, 35). The reliability of rPPG-based HRV therefore depends on experimental context, camera type, measurement duration, and signal-processing strategies.

### 2.4 Relevance of rPPG for stress monitoring

Because stress monitoring in real-world settings requires unobtrusive sensing, rPPG offers several advantages over contact PPG and ECG: no physical discomfort, no requirement for sensor placement, and direct integration with existing devices such as webcams and smartphones. Recent advances in algorithmic robustness and deep learning have allowed rPPG systems to detect stress, mental workload, and emotional arousal across diverse contexts including academic tasks (17), online meetings (18), sports anxiety (22), and social stressors (10). These developments position rPPG as a promising tool for scalable, continuous mental-state monitoring. Nevertheless, challenges related to HRV fidelity, environmental variability, and dataset heterogeneity limit generalizability and motivate the need for a focused, structured synthesis of existing work.

## 3 Literature search and selection method (PRISMA-ScR guided)

We conducted a scoping review to map and synthesize research on remote photoplethysmography (rPPG) for stress, mental workload, arousal, and closely related mental-state constructs. Given the methodological heterogeneity across computer vision, biomedical sensing, and affective computing, a scoping approach was selected rather than a meta-analytic systematic review. The search and selection process is reported following PRISMA-ScR guidance for transparency and reproducibility.

### 3.1 Information sources

We searched Scopus, IEEE Xplore, PubMed, and the ACM Digital Library as primary bibliographic databases. Google Scholar was used as a supplementary search engine to identify potentially relevant items not consistently indexed across databases. No study was included solely based on discovery via Google Scholar; all included studies met full-text eligibility criteria and had traceable publication metadata.

### 3.2 Full search strings and dates

Searches were executed during March 2026 (16–31. 2026). For each database, queries combined (i) at least one rPPG term, (ii) at least one stress/workload term, and (iii) optionally a physiology term (HRV/PRV). Minor syntax variations were applied to match each platform’s search interface.

**Scopus:**

(*“remote photoplethysmography”* OR *rPPG* OR *“imaging photoplethysmography”* OR *“video PPG”* OR *“camera-based PPG”* ) AND (*stress* OR *“mental workload”* OR *“cognitive load”* OR *anxiety* OR *arousal*) AND (*“heart rate variability”* OR *HRV* OR *“pulse rate variability”* OR *PRV* OR *“heart rate”*) AND PUBYEAR > 2009 AND PUBYEAR < 2027.

**IEEE Xplore:**

(*“remote photoplethysmography”* OR *rPPG* OR *“imaging PPG”* OR *“video PPG”* OR *“camera-based PPG”*) AND (*stress* OR *“mental workload”* OR *“cognitive load”* OR *anxiety* OR *arousal*) AND (*HRV* OR *“heart rate variability”* OR *PRV* OR *“pulse rate variability”*).

**PubMed:**

(*“remote photoplethysmography”* OR *rPPG* OR *“imaging photoplethysmography”* OR *“video PPG”*) AND (*stress* OR *“mental workload”* OR *“cognitive load”* OR *anxiety* OR *arousal*) AND (*HRV* OR *“heart rate variability”* OR *PRV* OR *“pulse rate variability”*).

**ACM Digital Library:**

(*“remote photoplethysmography”* OR *rPPG* OR *“imaging PPG”* OR *“video PPG”*) AND (*stress* OR *“mental workload”* OR *“cognitive load”* OR *anxiety* OR *arousal*) AND (*HRV* OR *“heart rate variability”* OR *PRV* OR *“pulse rate variability”*).

**Google Scholar (Supplementary; year range filter 2010–2026):**

Google Scholar was used solely as a supplementary source to capture relevant records not consistently indexed across the four primary databases. Three targeted queries were used: (1) *“remote photoplethysmography” stress HRV*; (2) *rPPG “mental workload” HRV*; and (3) *“imaging PPG” “cognitive load”*. Due to the high volume of automated returns, results were sorted by default relevance and a hard stopping rule was applied wherein only the first 200 records (20 pages) per query were screened. All records identified as potentially relevant during this supplementary screening were subjected to the same full-text eligibility assessment applied to primary database records. No study was included solely on the basis of Google Scholar discovery; all included studies have traceable DOIs and publication metadata.

### 3.3 Screening process, de-duplication, and record-type cleanup

**Figure 1:**
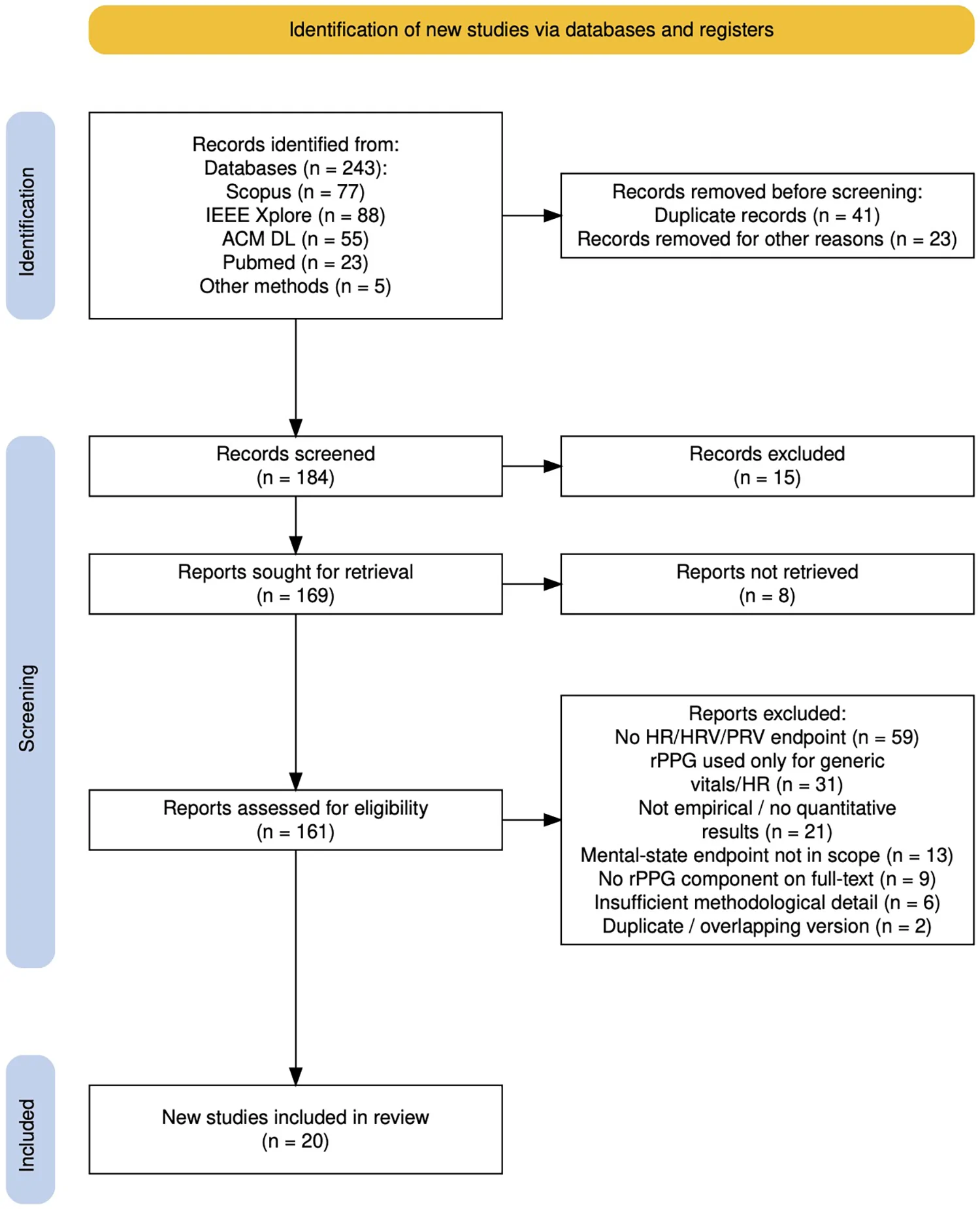
PRISMA-ScR flow diagram(PRISMA 2020 format) for study selection.

All retrieved records were exported into a reference manager and de-duplicated using DOI matching and title-based fuzzy matching, followed by manual verification for near-duplicates. As a record-type cleanup step prior to title/abstract screening, we removed non-study bibliographic artefacts (n=23). These included proceedings-level front-matter entries, editorials, and records without associated study content. They are reported in the PRISMA flow diagram (Figure 1) as “records removed for other reasons” and did not involve substantive content assessment.

Three reviewers participated in study selection. One reviewer conducted the primary title/abstract screening and full-text eligibility assessment across the complete dataset. The second and third reviewers each independently screened and verified 50% of records. Disagreements were resolved through discussion and consensus among all three reviewers. Reviewers were not blinded to authors, institutions, or publication venues; blinding is not required under PRISMA-ScR guidance for scoping reviews, and the exploratory mapping aim of this review does not depend on effect-size estimation for which blinding is methodologically relevant. No formal inter-rater agreement statistic (e.g., Cohen’s kappa) was calculated, consistent with PRISMA-ScR practice for scoping reviews where the aim is comprehensive mapping rather than confirmatory synthesis. The study selection process is summarized in the PRISMA flow diagram (Figure 1).

### 3.4 Screening and selection procedure (three-stage filtering)

To manage the breadth of retrieved literature, we applied a three-stage filtering procedure aligned with PRISMA-style phases (identification → screening → eligibility → inclusion). After de-duplication and record-type cleanup, filtering proceeded as follows:

**Stage 1: rPPG and Imaging-PPG identification (screening).** Records were retained if they explicitly used camera-based photoplethysmography (rPPG/imaging PPG/non-contact PPG) from RGB, infrared, or depth video. Works focusing exclusively on contact PPG (wearables, finger clips) or other sensing modalities (e.g., EEG-only, EDA-only, thermal-only) without an rPPG component were excluded.

**Stage 2: Stress/workload/arousal relevance (screening).** Titles and abstracts were screened for explicit linkage to stress, mental workload/cognitive load, anxiety, emotional arousal, or closely related constructs. Studies that used rPPG only for generic vital-sign estimation (e.g., heart-rate tracking without a mental-state endpoint) were excluded. Early rPPG stress works (14–16) were used as anchors for citation chaining at this stage.

**Stage 3: HR/HRV/PRV endpoints and validation (eligibility).** Full texts were assessed to confirm that papers reported quantitative HR/HRV/PRV (or closely related variability metrics) and used these measures for stress/workload inference, statistical association, or benchmarking. Validation against ECG or high-quality contact PPG was recorded as an indicator of evidential strength; wearable comparisons were treated as supportive rather than definitive when HRV fidelity was central.

### 3.5 Eligibility criteria

Studies were included if they:

- Used rPPG/imaging PPG/camera-based PPG from video (face or exposed skin),
- Extracted HR and/or HRV/PRV (or equivalent pulse-variability measures),
- Targeted stress, mental workload, arousal, anxiety, or closely related constructs,
- Reported empirical results (statistical analysis, classification/regression performance, or validation outcomes).

Studies were excluded if they:

- Relied exclusively on contact sensing (wearable PPG/ECG) without an rPPG component,
- Focused only on generic HR estimation without any mental-state relevance,
- Used only non-rPPG modalities (e.g., EEG-only, EDA-only, thermal-only) without rPPG-derived physiology,
- Lacked sufficient methodological detail to interpret findings (unless widely cited or foundational, in which case limitations were noted).

### 3.6 PRISMA counting rule and use of background references

Only empirical studies that used rPPG or imaging photoplethysmography from video and explicitly targeted stress, mental workload, anxiety, or arousal as an outcome were counted as included studies in the PRISMA-ScR synthesis (n=20).

Foundational rPPG algorithm papers, datasets and benchmarking papers, bias and fairness analyses, wearable-only physiological studies, and rPPG validation papers without a mental-state endpoint were retained as background references to support methodological context and discussion, but were explicitly excluded from PRISMA counts.

### 3.7 Data charting and extraction

For each included study, we extracted standardized fields to support cross-study synthesis:

- **Context and protocol:** setting (lab/field/telehealth/online), task type (e.g., mental arithmetic, Stroop-like, social-evaluative), duration, and labeling strategy (protocol blocks vs. self-report).
- **Acquisition:** camera type (webcam/RGB/IR/depth), frame rate/resolution (if reported), lighting conditions, and compression/streaming (if applicable).
- **rPPG pipeline:** ROI strategy, extraction approach (e.g., ICA/CHROM/POS/deep learning), filtering and motion handling, and any signal-quality measures.
- **Physiology and inference:** HR/HRV/PRV features, window length, model type (features+ML vs. end-to-end DL), and evaluation protocol (e.g., subject-wise cross-validation).
- **Validation:** reference sensors (ECG/contact PPG/wearables), agreement metrics (when provided), and robustness analyses (motion/lighting).

### 3.8 Synthesis approach

We performed a structured narrative synthesis organized around: (1) rPPG signal processing and estimation methods, (2) empirical evidence for stress and mental workload inference, (3) comparisons against contact PPG, wearables, and other modalities, and (4) recurring limitations and reporting gaps that affect robustness and generalization. References not meeting inclusion criteria were retained to support background, methodological context, and discussion, but were not counted as included studies. A brief justification for inclusion of each PRISMA-counted study is provided in the supplementary extraction table.

## 4 rPPG signal processing and estimation methods

Remote photoplethysmography (rPPG) estimates cardiovascular signals by analyzing subtle color variations in skin regions captured by cameras. Unlike contact photoplethysmography, rPPG is highly sensitive to environmental conditions and user motion, making signal processing a critical component for reliable heart rate (HR) and heart rate variability (HRV) estimation. This section reviews the main stages of rPPG signal processing, including signal acquisition, classical extraction methods, learning-based approaches, and the implications of these techniques for stress and mental workload assessment.

### 4.1 Signal acquisition and preprocessing

Most rPPG pipelines begin with face detection and region-of-interest (ROI) selection, typically focusing on areas such as the forehead or cheeks where skin exposure is high and motion is relatively limited. Pixel intensities within the ROI are spatially averaged to reduce sensor noise, followed by temporal detrending and normalization to suppress slow illumination changes. Motion artifacts arising from head movements, facial expressions, and camera jitter remain a primary challenge, often necessitating additional filtering or adaptive preprocessing (36, 37).

Environmental factors such as lighting intensity, spectral composition, camera frame rate, and compression artifacts significantly influence rPPG signal quality. Studies have shown that insufficient illumination or rapid lighting variation can distort pulse waveforms, while low frame rates limit the temporal resolution required for accurate HRV estimation (5, 34). These constraints motivate careful preprocessing and algorithm selection, particularly for stress-related applications where HRV sensitivity is critical.

### 4.2 Classical rPPG extraction methods

Early rPPG methods relied on simple color-channel analysis, exploiting the observation that green-channel intensity variations are strongly correlated with blood volume changes. While computationally efficient, green-channel approaches are highly susceptible to motion and illumination artifacts. To address these limitations, blind source separation techniques such as independent component analysis (ICA) were introduced, enabling separation of pulsatile and non-pulsatile components from RGB signals (6, 7).

Chrominance-based methods represent a major advance in rPPG robustness. The CHROM algorithm (8) projects RGB signals into a chrominance subspace designed to amplify the pulsatile component while suppressing motion-induced color changes. The POS method (9) further improves performance by adaptively normalizing color signals over short temporal windows. These methods remain widely used benchmarks due to their balance of computational simplicity and robustness under moderate motion and lighting variation.

Despite their success, classical methods exhibit limitations for stress analysis. Their performance degrades under large head movements, occlusions, and dynamic lighting, and they often underestimate high-frequency HRV components critical for stress detection (34, 35). As a result, classical rPPG approaches are typically more reliable for HR estimation than for fine-grained HRV analysis.

Following pulse waveform extraction, HR and HRV estimation requires accurate peak detection and inter-beat interval computation. Time-domain HRV metrics such as RMSSD and SDNN are commonly used due to their relative robustness to short measurement windows, whereas frequency-domain features (e.g., LF, HF, LF/HF ratio) are more sensitive to noise and require longer, stationary signals.

Multiple studies report that rPPG-derived pulse rate variability (PRV) correlates with ECG-based HRV under controlled conditions, but exhibits systematic bias and reduced reliability under motion or naturalistic behavior (36, 51). Ultra-short-term HRV estimation (e.g., 20–60 s), frequently employed in stress detection systems, further amplifies these limitations, leading to underestimation of variability and increased false positives (37, 44, 52). These findings have direct implications for stress monitoring, where transient autonomic changes are of primary interest.For quick benchmarking context, Table 2 lists commonly used datasets and validation anchors referenced throughout this review.

**Table 2:** Key datasets and validation anchors cited for benchmarking context (background references; excluded from PRISMA synthesis).

Dataset/Source	Modality	Labels/Task	Reference sensor	Notes (relevance)
UBFC-Phys (45)	Face video + contact signals	Social stress (TSST-inspired)	Wrist contact BVP + EDA	Designed for psychophysiological stress; enables contactless vs. contact comparisons. Also counted as PRISMA-included study P16 (reports rPPG-based stress recognition results, 85.48% accuracy).
UBFC-rPPG (40)	Face video	Vitals benchmark	Dataset-provided contact reference	Widely used rPPG benchmark; useful for validating extraction methods under motion/illumination changes.
COHFACE (46)	Face video	Vitals benchmark	Dataset-provided contact reference	Common public benchmark for algorithm validation and cross-paper comparability.
VIPL-HR (47)	Face video (less constrained)	Vitals benchmark	Dataset-provided contact reference	Useful for evaluating robustness beyond tightly controlled recordings.
MMPD (48)	Mobile face video	Vitals benchmark	Dataset-provided contact reference	Multi-domain mobile capture; relevant for cross-device and cross-context testing.
DIB-10min (49)	Long face video (uncontrolled lighting)	Vitals benchmark	Dataset-provided contact reference	Long-duration video can stress-test drift, lighting variation, and SQI-gated inference behavior.
SWELL-KW (50)	Workplace physiology (no video)	Stress/workload conditions	ECG (dataset-provided)	Stress/workload anchor dataset; useful for protocol design and feature selection, even without rPPG.

### 4.3 Learning-based rPPG methods

Recent advances in machine learning and deep learning have enabled end-to-end rPPG signal extraction and stress inference. DeepPhys (10) introduced convolutional attention mechanisms to learn spatial and temporal representations of pulse signals directly from video. Subsequent works employ convolutional neural networks (CNNs), recurrent architectures (LSTM, GRU), and multi-task learning to jointly estimate HR, HRV, and stress-related features (26, 27). Learning-based reconstruction of rPPG waveforms from facial video has also been explored to improve waveform fidelity for downstream analysis (53).

Learning-based approaches offer improved robustness to noise and can implicitly model complex motion patterns. However, they require large, diverse datasets and are prone to overfitting when trained on small or homogeneous samples. Deep models also often act as black boxes, complicating interpretability and physiological validation, which is an important consideration for stress assessment systems (28). As a result, many studies combine learning-based extraction with classical preprocessing to balance robustness and transparency. Open-source implementations and toolboxes can support reproducibility and fair comparison across methods (54–56).

### 4.4 Implications for stress and mental workload detection

The signal processing characteristics of rPPG impose fundamental constraints on stress and mental workload detection. While rPPG can reliably capture HR and coarse autonomic trends, HRV estimation remains sensitive to motion, lighting, and short recording windows. Consequently, stress detection systems based on rPPG often rely on a combination of time-domain HRV features, trend-based analysis, and machine learning classifiers rather than precise frequency-domain metrics.

These limitations highlight the importance of careful validation against ECG, contact PPG, or wearable devices when evaluating rPPG-based stress systems. Understanding the signal processing trade-offs reviewed in this section is essential for interpreting the empirical findings presented in subsequent sections on rPPG-based stress and workload assessment.

## 5 rPPG for stress and mental workload assessment

This section synthesizes empirical evidence on the use of rPPG-derived cardiovascular signals for stress, mental workload, anxiety, and arousal assessment. Rather than summarizing individual studies in isolation, this section synthesizes the PRISMA-included empirical literature (n=20) along validity-critical dimensions that determine whether rPPG-derived cardiovascular signals can support defensible stress or workload inference. These dimensions include outcome construct, window length, reference modality, evaluation protocol, and deployment context. We focus on studies that (i) employ rPPG or imaging PPG to extract HR/HRV/PRV, and (ii) evaluate stress- or workload-related outcomes through classification, regression, or statistical association. Across the literature, rPPG is typically used as a non-contact proxy for autonomic activity and is often compared against ECG, contact PPG, or wearable measurements to validate physiological fidelity.

### 5.1 Validity of rPPG-based stress inference

Remote photoplethysmography (rPPG)–based stress and mental workload assessment relies on the assumption that cardiovascular markers extracted from facial video reflect autonomic nervous system activity. While this assumption is well supported for heart-rate estimation under controlled conditions, its validity becomes less clear when extended to heart rate variability (HRV), pulse rate variability (PRV), and downstream stress inference, particularly under short analysis windows and realistic acquisition constraints.

Across the reviewed literature, stress inference is operationalized using heterogeneous targets and validation strategies. Some studies infer stress directly from protocol-defined task blocks or dataset labels, others incorporate subjective self-reports, and a smaller subset validates rPPG-derived signals against electrocardiography (ECG) or contact photoplethysmography (PPG). As a result, reported stress-classification performance spans a wide methodological spectrum, ranging from indirect, protocol-driven inference to physiologically anchored validation. These differences substantially affect the interpretability and comparability of reported results.

A central challenge is that high stress-classification accuracy does not necessarily imply accurate recovery of autonomic dynamics. Protocol-driven stress induction often co-varies with motion, facial behavior, speech activity, or illumination changes, all of which can influence rPPG signal quality independently of physiological stress responses. In such cases, classification models may exploit task-correlated artifacts rather than genuine cardiovascular variability. This risk is amplified when HRV/PRV features are extracted from ultra-short windows and when wearable devices are used as reference sensors despite their own limitations under non-resting conditions.

**Figure 2:**
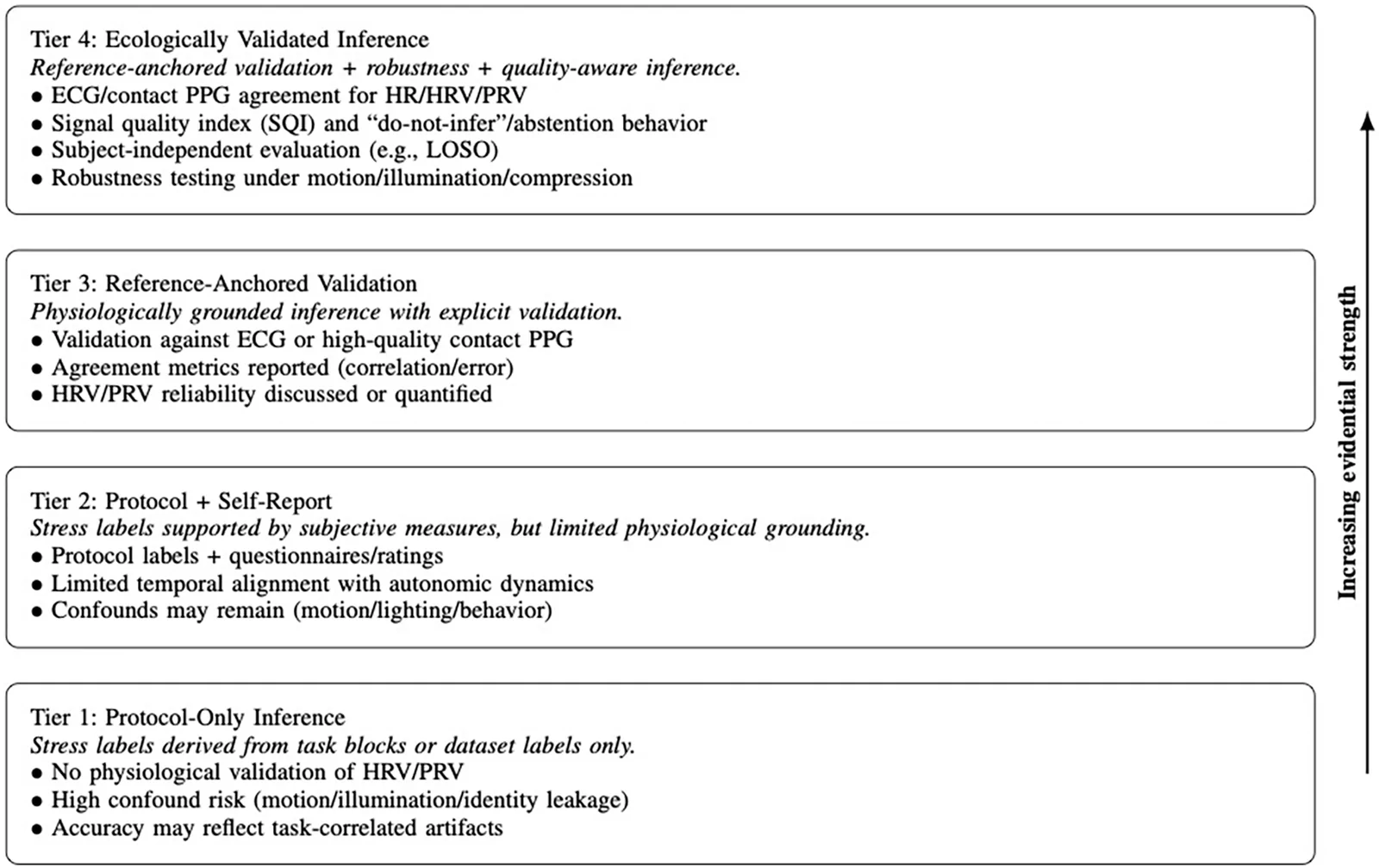
Validity ladder for rPPG-based stress and mental workload inference. Lower tiers rely primarily on protocol labels or self-report and are vulnerable to confounds unrelated to autonomic activity. Higher tiers incorporate reference-anchored physiological validation, signal-quality awareness, and robust evaluation protocols, enabling more defensible stress inference.

To address this heterogeneity, we introduce a validity-oriented framework that differentiates rPPG-based stress inference according to the strength of its physiological grounding and robustness to confounds. Figure 2 presents a validity ladder that categorizes studies based on their reliance on protocol labels, self-report, reference-anchored validation, and robustness measures such as signal-quality awareness and subject-independent evaluation, while Table 3 summarizes scenario-level defensibility across deployment contexts. Mapping the included studies onto this ladder reveals that most rPPG-based stress and workload systems operate in the lower-to-middle tiers of evidential strength, typically relying on protocol-defined labels and short analysis windows, with limited reference-anchored physiological validation. Only a small subset of studies combine rPPG with ECG or high-quality contact PPG, subject-independent evaluation, and explicit signal-quality handling, corresponding to the higher tiers of the ladder. Table 3 complements this framework by summarizing when rPPG-based stress inference is physiologically defensible across common experimental and deployment scenarios. Together, these tools provide a structured basis for interpreting existing evidence and for guiding future rPPG-based stress research toward more reliable and generalizable outcomes.

**Table 3:** Scenario-level validity matrix for rPPG-based stress inference.

Scenario/Context	Observed window (representative studies)	Primary target	Reference used	Verdict	Explicit rationale + key studies
Lab, seated, low motion	≥60 s moving window (14); 11-min protocol (57)	HR (and trends)	ECG/contact PPG	Defensible	ECG/PPG agreement demonstrated. HR stable; HRV feasible if stationarity holds. (14): HR r=1.0; (57): SDNN PCC = 0.90.
Lab stress tasks (cognitive/social)	30 s window (16); ∼1 min (15, 20, 22); 2 min (29)	HRV/PRV	None / labels-only	Conditional	Short-window HRV/PRV instability; protocol co-varies with motion. Defensible only with SQI gating + contact reference. (15, 16, 20, 29).
Academic workload settings	NR (17)	HR + time-domain HRV	Wearable PPG	Conditional	Uncontrolled environment; questionnaire labels; no cardiac reference. (17). Requires SQI + reference validation.
Online meetings/speaking interaction	5 min SNR-gated segments (18)	HR + coarse HRV	Contact PPG (when available)	Conditional	Speech, facial motion, camera auto-exposure. (18) mitigates via SNR gating + subject-independent CV. Without these safeguards: Not defensible.
Telehealth livestream / compressed video	NR (19) (framework only)	HR (primarily)	Contact PPG (preferred)	Conditional	(19) is a framework paper with no empirical validation. Compression/bitrate variability; requires robustness analysis before inference.
In-the-wild webcam monitoring	NR (22, 30)	HRV/PRV	None	Not defensible	No physiological reference; no SQI; condition-correlated confounds dominate. Limit to HR/coarse trends with abstention. (22, 30).
Multimodal + SQI-gated systems	5 min (18); quality-gated (31); BVP-rPPG filter (32)	HR + validated HRV features	ECG/contact PPG	Most defensible	ECG/contact PPG + SQI/quality gating + subject-independent evaluation. (14, 18, 31, 32).

“Defensible” = inference likely reflects autonomic changes under stated conditions. “Conditional” = defensible only when additional safeguards (SQI gating, physiological reference, subject-independent evaluation) are applied. “Not Defensible” = high risk that inference is driven by confounds or unreliable HRV/PRV estimates. Window lengths are from representative included studies; NR = not reported in the original paper.

#### 5.1.1 Measurement validity vs. construct validity

It is important to distinguish two dimensions of validity when evaluating rPPG-based stress inference. *Measurement validity* concerns whether rPPG accurately recovers the cardiovascular signals of interest, that is, whether rPPG-derived HR, HRV, or PRV agrees with reference measurements from ECG or high-quality contact PPG. The validity ladder (Figure 2) primarily operationalises this dimension. Studies at higher tiers provide stronger evidence of physiological signal fidelity. However, even perfect measurement validity does not guarantee *construct validity*, which is the assumption that the cardiovascular marker, however accurately captured, meaningfully reflects the target psychological construct (stress or workload) rather than a confounding process. In rPPG research, construct validity is most threatened when stress protocols co-vary with motion, speech, or illumination changes that independently alter signal quality or HR. Studies that report high stress-classification accuracy without controlling for such condition-correlated confounds may achieve good measurement-level agreement while suffering poor construct validity. We recommend that future studies address both dimensions explicitly: report physiological agreement against a reference (measurement validity), and evaluate whether classification performance holds after controlling for task-correlated confounds (construct validity).

#### 5.1.2 Quantitative synthesis across validity dimensions

Across the 20 PRISMA-included studies, reference sensor usage was distributed as follows: 1 study used ECG or ECG-based dataset references (31); 1 study used Holter ECG for beat-timing validation (21); 5 studies used non-wrist contact PPG [finger oximeter or validated contact PPG: (14, 18, 25, 29, 57)]; 5 studies used wristband BVP via the UBFC-Phys dataset [Empatica E4: (26–28, 32, 45)]; 2 studies used EDA as the sole physiological comparator with no cardiac reference (15, 16); and 6 studies reported no physiological reference sensor (17, 19, 20, 22, 30, 52). Agreement metrics (correlation, MAE, or Bland-Altman) were explicitly reported in 5 studies (14, 18, 31, 52, 57). Subject-independent evaluation was explicitly reported in 5 studies (14, 18, 27, 32, 52). Robustness testing under motion, illumination, or streaming artefacts was explicitly reported in 4 studies (18, 25, 29, 52), with qualitative mention in 3 further studies (15, 16, 21).

### 5.2 Experimental paradigms and ground truth

Stress and workload studies employing rPPG commonly use controlled cognitive tasks (e.g., mental arithmetic, Stroop-like protocols, or time-pressure tasks), social-evaluative stressors, and academic workload settings. Ground truth labels are obtained either through (i) protocol-driven condition labels (baseline vs. stress blocks), (ii) self-report measures (e.g., perceived stress ratings), or (iii) concurrent physiological reference sensors. While protocol labels are widely used, physiological validation against ECG/contact PPG is especially important when HRV is a primary endpoint, given the sensitivity of HRV/PRV to motion and illumination artifacts (34, 35, 52).

### 5.3 Physiological features and modeling approaches

Most rPPG stress systems extract a combination of heart rate, pulse morphology proxies, and HRV/PRV features. Time-domain variability measures (e.g., RMSSD, SDNN) are frequently reported due to better stability under short measurement windows, whereas frequency-domain metrics are less consistently reliable unless recordings are long and stationary (36, 51). Modeling approaches range from statistical comparisons (baseline vs. stress), to classical machine learning classifiers using HRV features, to end-to-end deep learning pipelines that learn stress-relevant representations from video (10, 26–28).

A practically important question for study design is whether HRV features provide incremental predictive value for stress/workload classification beyond heart rate (HR) alone. Direct ablation evidence within the PRISMA-included studies is limited: only McDuff et al. (14) explicitly tested HR as an isolated feature and found it the least discriminative single predictor (SVM accuracy: HR alone 60% vs. combined HRV and breathing rate 85%), while HRV LF, HF, and breathing rate drove classification performance. Most other included studies report combined HR+HRV feature sets without isolating individual contributions, and end-to-end deep learning studies (26–28) do not separate feature contributions by design. The broader stress physiology and machine-learning literature supports the expectation that HRV carries incremental specificity: meta-analytic evidence confirms that HF-HRV and RMSSD specifically decrease under psychological stress while HR elevates non-specifically under both physical and psychological load (42). In machine-learning benchmarks using ECG-derived HRV, combined time- and frequency-domain HRV features substantially outperform heart-rate-only approaches for multi-class stress classification (58, 59). Within rPPG systems, this incremental advantage is conditional on signal quality: when rPPG-derived HRV is degraded by motion or short windows (36, 52), HR becomes the more reliable endpoint and the HRV advantage likely diminishes or reverses. Feature ablation experiments isolating rPPG HR vs. HRV contributions across deployment conditions remain a priority for future research.

### 5.4 Performance trends and reliability considerations

Early studies demonstrated feasibility of detecting cognitive stress using rPPG-derived HRV/PRV signals (14–16). More recent work reports high classification performance, particularly when deep learning is applied to controlled datasets (25–27). However, reported accuracies are not directly comparable across studies due to differences in camera hardware, sampling rates, ROI strategies, preprocessing, stress protocols, and label definitions. The differential reliability of rPPG for HR vs. HRV is supported directly by agreement data from several included studies. McDuff et al. (14) reported near-perfect HR agreement against contact PPG (r=1.0) alongside HRV LF and HF correlations of r=0.93 under well-controlled laboratory conditions. This is a best-case scenario that illustrates the ceiling achievable with high-quality hardware. Odinaev et al. (CHASE 2022) (57) reported SDNN PCC of 0.90 against a contact pulse oximeter during Stroop stress tasks, but RMSSD PCC of only 0.45 and LF/HF PCC of only 0.40. This demonstrates that variability metrics are substantially less reliable than SDNN even under the same reference and protocol. Liu et al. (31) reported HR correlation r=0.86 against ECG but substantially lower HRV agreement (SDNN r=0.32, RMSSD r=0.24, pNN50 r=0.25) after quality gating. Sun et al. (18) reported HR MAE of 5.13 bpm against contact PPG in realistic online meeting conditions, without reporting separate HRV agreement. Together, these findings confirm that rPPG HR estimation is substantially more reliable than HRV/PRV estimation. The gap is most pronounced for parasympathetically sensitive metrics (RMSSD, HF-HRV) and under conditions involving motion, short windows, or naturalistic capture (34, 37, 52).

Table 4 highlights twelve studies selected from the PRISMA-included set (n=20) to illustrate the diversity of methodological patterns and validity limitations. Studies were chosen to represent: (i) all four validity ladder tiers; (ii) both classical and deep-learning rPPG method types; (iii) multiple deployment contexts (laboratory, online, telehealth); and (iv) the range of reference sensor strength from ECG/contact PPG to wearable to none. The complete data extraction for all 20 studies is provided in Supplementary Table S1.

**Table 4:** PRISMA-included rPPG-based stress and workload studies illustrating key methodological patterns and validity limitations across all four validity ladder tiers.

Study	Context/Task	Subject-independent evaluation (explicitly reported?)	rPPG Method	Ground truth	Key outcome
McDuff et al. (14)	Cognitive stress tasks	Yes (person-independent)	Classical rPPG + HRV/PRV	Protocol labels; physiological reference	Demonstrated feasibility of remote stress inference using HRV-derived features under controlled cognitive stress.
Bousefsaf et al. (15, 16)	Mental stress/workload paradigms	Not reported	rPPG-based HRV/PRV extraction	Protocol labels; validation reference	Early evidence that webcam-derived HRV can separate stress from baseline under controlled paradigms.
Nikolaiev et al. (21)	Stress index estimation	Not reported	rPPG-derived stress index formulation	Protocol labels	Proposed a non-contact stress index computed from rPPG features, relying on protocol-driven labels.
Chen et al. (20)	Mental arithmetic stress	Not reported	rPPG-based HRV measurement + classifier	Protocol labels; reference sensors	Demonstrated HRV-based stress detection under controlled task induction with limited ecological validity.
Iuchi et al. (22)	Stress level estimation	Not reported	PRV from facial rPPG	Protocol labels/self-report	Reported stress-level separability using facial PRV features, sensitive to acquisition conditions.
Morales-Fajardo et al. (17)	Academic stress environment	Not reported	rPPG + HRV/PRV with ML	Protocol labels / self-report	Reported stress identification in academic-like settings, highlighting inter-subject variability.
Sun et al. (18)	Online meetings	Yes (subject-independent, 10-fold CV)	rPPG combined with behavioral cues	Self-report / protocol	Demonstrated improved robustness in realistic interaction settings when rPPG is combined with contextual features.
Xu et al. (27)	Non-contact stress recognition	Yes (LOSO, explicitly reported)	Multi-task deep learning on rPPG	Dataset labels / protocol	Reported strong performance using representation learning; interpretation sensitive to labeling and splits.
Fontes et al. (26)	Stress detection benchmarks	Not reported	rPPG with deep learning	Dataset labels	Reported performance gains using deep learning on rPPG signals under benchmarked datasets.
Song and Lee (44)	Ultra-short stress measurement	Not reported	rPPG + PRV analysis	Protocol labels; validation reference	Explicitly quantified limitations of ultra-short temporal windows, motivating “do-not-infer” conditions for PRV-based stress inference.
Odinaev et al. (57)	Cognitive stress (Stroop, 11 min)	Not reported	POS + wavelet bandpass + IBI analysis	Contact PPG (pulse oximeter)	Reported HRV agreement vs. contact PPG under stress: SDNN MAE 4.89 ms (PCC 0.90); RMSSD PCC 0.45; LF/HF PCC 0.40, demonstrating differential HR vs. HRV reliability.
Du and Yang (30)	Stress classification (dual-modality)	Not reported	Uncertainty-driven fusion (pulse + facial image features)	Protocol labels only	Achieved 80.13% accuracy using multimodal fusion; no physiological reference sensor; illustrates Tier 1 protocol-only inference.

Note: Only studies explicitly reporting subject-independent evaluation are labelled accordingly; all others are marked “Not reported”. The complete PRISMA-included set (n=20) is provided in Supplementary Table S1.

### 5.5 Key gaps and reporting recommendations

Based on the reviewed literature, three recurring issues limit reproducibility and deployment:

- **Protocol and labeling variability:** Stress induction paradigms and label definitions differ substantially across studies, complicating cross-paper comparisons.
- **Insufficient validation for HRV/PRV:** Many works report stress classification performance without adequately validating the fidelity of rPPG-derived HRV/PRV against ECG or contact PPG, despite known sensitivity to motion and lighting (34, 52).
- **Limited reporting of acquisition conditions:** Camera parameters (frame rate, resolution), illumination conditions, ROI strategy, and motion handling are often underreported, yet strongly determine HRV reliability and stress inference stability (36, 37).

To improve comparability, future work should report acquisition settings, provide explicit ground-truth references when claiming HRV-based inference, and evaluate robustness under realistic motion and lighting conditions. These requirements are particularly important for stress and workload monitoring in everyday settings, where uncontrolled variability dominates. Based on the synthesis, several do-not-infer conditions emerge. In particular, rPPG-based stress or workload inference should be avoided when (i) analysis windows are ultra-short and dominated by motion or illumination changes, (ii) no physiological reference is available to validate HRV or PRV fidelity, or (iii) signal quality cannot be monitored and low-quality segments cannot be excluded or flagged. In such cases, reporting heart rate or coarse autonomic trends, or abstaining from inference altogether, is more defensible than attempting HRV- or PRV-based stress estimation.

Complementing the do-not-infer conditions above, we propose the following minimum requirements for defensible rPPG-based stress or workload inference: (1) **Window length:** analysis windows should be at least 120 s for time-domain HRV metrics (RMSSD, SDNN); windows shorter than 60 s should restrict inference to HR and coarse autonomic trends only (36, 44). (2) **Signal quality:** a signal-quality index (SQI) or SNR threshold should be applied before HRV/PRV extraction (60); segments below threshold should be excluded or flagged, and abstention from inference triggered when the proportion of usable segments is insufficient. (3) **Reference validation:** when HRV or PRV features drive stress inference, at least one study-phase comparison against ECG or high-quality contact PPG should be reported; wristband BVP is acceptable as a practical baseline but should be acknowledged as a limited ground truth under non-resting conditions (1, 2). (4) **Evaluation protocol:** classifiers should be evaluated using subject-independent splits (LOSO or stratified k-fold with subject exclusion) to prevent identity leakage from inflating reported performance (14, 18, 27). (5) **Label quality:** protocol block labels are acceptable for exploratory studies but should be complemented by validated subjective measures or concurrent physiological markers when claims about autonomic stress responses are made. These represent a defensible minimum for translatable inference, not absolute thresholds.

## 6 Comparison with contact PPG, wearables, and other modalities

A recurring question in the rPPG stress literature is whether camera-based pulse measurements can serve as a practical substitute for contact-based sensing (ECG/contact PPG) or consumer wearables, particularly when stress inference depends on HRV/PRV features. This section summarizes comparative evidence and highlights when rPPG is a reasonable choice, when it is not, and what validation practices are necessary for credible stress/workload claims.

### 6.1 Comparison dimensions

Across studies, rPPG and contact modalities differ along several technically relevant dimensions:

**Signal fidelity:** ECG provides the most reliable inter-beat intervals for HRV analysis, while contact PPG can be reliable but is sensitive to motion and contact conditions. Wrist-worn wearable PPG is particularly vulnerable to motion artifacts and fit variability, which can degrade HRV/PRV reliability during non-resting or stress-inducing tasks (1, 2).

**Environmental sensitivity:** rPPG performance depends strongly on illumination, camera quality, compression/streaming artifacts, and facial motion. In contrast, contact modalities depend more on sensor placement, contact pressure, and peripheral perfusion.

**User burden and scalability:** rPPG reduces user burden because it is non-contact and can be integrated into existing cameras (phones, laptops, telehealth systems). However, non-contact sensing introduces additional uncertainty that must be managed via robust signal-quality handling and appropriate reporting (36, 37).

When the primary objective is accurate HRV, ECG remains the preferred benchmark. rPPG has demonstrated strong feasibility for heart rate and coarse autonomic trends, but HRV/PRV estimation is generally less stable under motion and short windows. Validation studies comparing imaging PPG with wearable monitoring highlight that agreement can be acceptable under controlled conditions but deteriorates as motion and environmental variability increase (34, 35). Similarly, feasibility work on ultra-short windows indicates that PRV reliability is strongly dependent on signal quality and recording constraints (36).

For stress/workload assessment, this implies a key methodological requirement: *if an rPPG system claims HRV-driven stress inference, it should report physiological agreement against ECG or high-quality contact PPG whenever possible*. In the absence of such validation, performance numbers based solely on protocol labels can be misleading because stress classification may inadvertently exploit dataset artifacts (e.g., lighting or motion differences between conditions) rather than true autonomic changes. This risk is amplified by known demographic and skin-type biases in remote physiological sensing and by bias in public datasets (61–63).

### 6.2 rPPG vs. wearable PPG

Wearable PPG is often used as a practical reference modality because it is widely available. However, wearable HRV/PRV itself can be unreliable in stress contexts, especially at the wrist, making it an imperfect gold standard. Studies assessing PRV from wrist-worn devices suggest limitations in using wearable PRV as a direct surrogate for ECG-based HRV (1), and acute stress detection under non-resting conditions can be challenging even for contact PPG, depending on motion and model choice (2). These findings complicate simple benchmarking narratives such as “rPPG matches wearables,” because wearables may not provide a stable reference under the very conditions where stress inference is most relevant.

In contrast, alternative form factors such as earbud PPG may provide stronger signal quality and richer morphological features, improving stress detection robustness in some settings (4). In addition, wearable-device pipelines for mental workload assessment demonstrate that good performance is achievable but remains sensitive to evaluation protocols, sensor conditions, and feature stability (38). On balance, the wearable literature reinforces the need to interpret rPPG comparisons carefully: wearables can be a useful baseline for practical deployment, but not necessarily a high-fidelity ground truth for HRV claims.

### 6.3 Comparison with other modalities

Many stress-monitoring systems use additional modalities (e.g., electrodermal activity, respiration, thermal imaging, behavioral cues). While these can enhance sensitivity, they also introduce fusion complexity and may shift the target from purely cardiovascular proxies to broader stress phenotyping. For rPPG-centered systems, the most relevant comparisons are those that preserve physiological interpretability and provide clear validation pathways. Studies incorporating complementary sensing (e.g., thermal with rPPG) show potential improvements for detecting subtle mental states, but careful ablation is required to isolate the contribution of rPPG-derived physiology (31).

### 6.4 Practical recommendations for benchmarking

Based on comparative evidence, we recommend the following benchmarking practices for rPPG-based stress and workload research:

- **Use ECG or validated contact PPG when HRV is central:** If HRV/PRV features drive the stress inference, include a physiological reference and report agreement (e.g., correlation, error statistics) rather than reporting only classifier accuracy (34, 35).
- **Report acquisition and quality controls:** Provide camera frame rate, resolution, lighting conditions, ROI strategy, motion handling, and any signal-quality indices, as these directly determine HRV/PRV stability (36, 37).
- **Interpret wearable comparisons cautiously:** Wrist-worn wearable PRV/HRV may be unstable during stress and motion; treat wearables as a practical baseline, not a definitive gold standard (1, 2).
- **Prefer subject-wise evaluation:** Stress models should be evaluated with subject-independent splits whenever possible to reduce identity leakage and inflate-free performance reporting.

In sum, rPPG offers a compelling non-contact sensing channel for stress and workload monitoring, especially in telehealth and camera-rich settings. However, credible claims require explicit validation, careful reporting of acquisition conditions, and conservative interpretation of HRV/PRV features under real-world variability.

From a system-design perspective, the reviewed evidence suggests the following rules of thumb: (i) HRV- or PRV-driven stress inference should be supported by ECG or high-quality contact PPG whenever feasible; (ii) wearable PRV should be treated as a practical baseline rather than a physiological gold standard; (iii) subject-independent evaluation is essential to reduce identity leakage and confounded performance; and (iv) signal-quality-aware pipelines with explicit abstention behavior are preferable to always-on inference.

## 7 Multimodal and real-world rPPG applications

Although many rPPG-based stress and workload studies are conducted in controlled settings, a growing subset targets more realistic usage scenarios and/or improves robustness through multimodal sensing. In practice, rPPG is most vulnerable to motion, illumination variation, and camera quality; therefore, real-world adoption often requires either (i) stronger artifact handling and validation, or (ii) complementary modalities that compensate when rPPG signal quality degrades.

### 7.1 Multimodal fusion for robust stress inference

Several studies indicate that combining rPPG with non-physiological cues (e.g., behavioral or contextual signals) can improve stress inference in interactive settings. For example, multimodal systems that integrate rPPG with behavioral indicators have demonstrated improved robustness in scenarios such as online meetings, where posture changes, speaking activity, and attention shifts can affect signal quality and stress expression (18). Similarly, fusion with thermal imaging has been explored to improve sensitivity to subtle mental states compared to unimodal rPPG, particularly when cardiovascular features alone are insufficient or unreliable (31). Recent deep learning approaches also increasingly incorporate multimodal inputs or fusion strategies to boost performance, though cross-context generalization remains inconsistently evaluated (27, 28).

### 7.2 Real-world and telehealth-oriented deployments

Real-world use cases motivate rPPG because it can be deployed with commodity cameras and minimal user burden. Telehealth-like or remote monitoring scenarios are frequently cited as promising applications, but they introduce additional constraints such as video compression, bandwidth variability, and uncontrolled lighting. Work targeting telehealth or remote-stream settings suggests feasibility, yet also underscores the need for careful validation and reliability assessment under realistic network and environment conditions (19). Across such deployments, HR estimation is typically more stable than HRV/PRV, and stress inference claims should be grounded in physiological fidelity checks where possible (34, 52).

### 7.3 Practical considerations for deployment

Across multimodal and real-world studies, three practical considerations recur. First, *signal quality monitoring* (explicitly measuring when rPPG is reliable vs corrupted) is essential for preventing spurious HRV/PRV features from driving stress inference. Second, *robustness testing* should include realistic motion and lighting variability, since controlled performance often overestimates field performance (36, 37). Third, *evaluation beyond single datasets* is increasingly important; models that perform well on a single benchmark may not transfer across devices, contexts, or demographics without adaptation (26, 27).

Taken in aggregate, the literature suggests that rPPG-based stress monitoring is most defensible in real-world settings when (i) multimodal inputs are used to improve robustness, (ii) physiological validation is performed for HRV/PRV-based claims, and (iii) acquisition and deployment constraints are explicitly reported and tested.

## 8 Discussion: limitations, open challenges, and future directions

The limitations discussed here reflect structural challenges inherent to camera-based cardiovascular sensing under realistic conditions, rather than deficiencies of individual studies. Although rPPG has matured substantially as a non-contact physiological sensing modality, the reviewed stress and mental workload literature exhibits recurring limitations that constrain reproducibility, comparability, and deployment confidence. This section consolidates the most critical technical challenges and outlines research directions that can meaningfully advance rPPG-based stress assessment.

### 8.1 Limitations of current evidence

**Heterogeneous stress protocols and labels.** A major barrier to synthesis is the lack of standardized stress/workload induction protocols and ground-truth definitions. Many studies rely on protocol block labels (baseline vs. stress) or coarse self-reports, while stress intensity and autonomic reactivity vary substantially across individuals. As a result, performance metrics across papers are often not directly comparable.

**HRV/PRV fidelity under realistic conditions.** While rPPG is often robust for heart rate estimation, HRV/PRV extraction is fragile under head motion, facial expressions, occlusions, dynamic lighting, and short-window recordings (36, 37, 52). Since stress inference frequently depends on subtle variability changes rather than mean HR alone, insufficient signal fidelity can lead to unstable or spurious inferences.

**Incomplete reporting of acquisition and preprocessing.** Many works underreport camera parameters (frame rate, resolution), illumination conditions, compression/streaming settings, ROI strategies, and motion-handling mechanisms. These details materially affect HRV/PRV outcomes and must be reported to interpret or reproduce results (36, 37). As summarized in Supplementary Table S1, most included studies do not explicitly report subject-independent evaluation protocols, highlighting a critical gap in generalization reporting within the rPPG-based stress literature.

**Evaluation pitfalls and generalization limits.** Reported classification accuracies may be inflated when evaluation protocols are not subject-independent or when confounding factors correlate with conditions (e.g., lighting differences between baseline and stress segments). In addition, models trained on constrained datasets may not generalize across cameras, settings, and populations. Dataset composition and demographic balance can materially affect reported performance and should be reported explicitly (61, 63). These issues are particularly relevant for deep learning approaches, which can learn shortcut cues unless robust evaluation and ablations are performed (27, 28).

### 8.2 Open challenges

**Signal quality assessment and “do-not-infer” behavior.** A critical requirement for practical stress monitoring is the ability to detect low-quality rPPG segments and suppress inference. Signal-quality indices tailored to rPPG can support this “do-not-infer” behavior (60). Without explicit signal-quality indices, systems risk producing confident stress outputs when the underlying physiological estimate is unreliable.

**Short-window variability estimation.** Many stress applications prefer short windows (e.g., under one minute) for responsiveness, yet ultra-short HRV/PRV estimation remains challenging and often underestimates variability (36). A key open question is which variability features remain valid under short, noisy recordings and how to quantify uncertainty.

**Cross-device and cross-context robustness.** Differences in camera hardware, optics, frame rates, and compression pipelines can substantially alter rPPG quality. Robust stress inference will require systematic cross-device benchmarking and adaptation strategies, rather than evaluation on a single camera/dataset.

**Reference-anchored validation for stress inference.** Comparisons against wearables are useful but insufficient when wearable PRV/HRV is itself unstable under motion and stress conditions (1, 2). For HRV-driven claims, ECG or validated contact PPG reference remains important for credible evaluation.

### 8.3 Future research directions

Based on the reviewed literature, we highlight five actionable research directions:

1. **Standardized reporting and benchmarking.** Future studies should report camera specifications (frame rate, resolution), lighting conditions, ROI selection, preprocessing, and window lengths for HRV/PRV computation. Shared benchmarking protocols across datasets and devices would substantially improve comparability.
2. **Joint physiological validation and stress modeling.** When stress inference relies on HRV/PRV, studies should include reference sensors (ECG or high-quality contact PPG) and report agreement measures (e.g., correlation and error metrics), not only downstream classification accuracy (34, 35).
3. **Robustness testing under motion and illumination.** Stress models should be evaluated under controlled perturbations (motion levels, lighting changes, compression/streaming artifacts) with explicit failure analyses. Reporting robustness curves rather than single accuracy values would better reflect real-world viability.
4. **Cross-device generalization and calibration.** To enable deployment, models should be tested across multiple cameras and capture conditions. Calibration or domain adaptation strategies may be necessary, particularly for deep learning models trained on limited data.
5. **Multimodal fusion and ablation-driven evidence.** Combining rPPG with complementary signals (e.g., thermal imaging or behavioral features) can improve sensitivity to mental states (18, 31), but future work should include clear ablations to isolate the contribution of rPPG-derived physiology and avoid confounded performance claims.

On the whole, the literature supports rPPG as a promising non-contact modality for stress and mental workload assessment in constrained settings. Advancing toward real-world monitoring requires rigorous validation, transparent reporting, and robustness-first evaluation practices that explicitly account for the fragility of short-window HRV/PRV estimation.

## 9 Conclusion

This review examined the state of remote photoplethysmography (rPPG) for stress and mental workload assessment, with emphasis on signal-processing pipelines, HR/HRV/PRV estimation reliability, and empirical evidence from stress/workload studies. Across the literature, rPPG consistently supports non-contact heart-rate monitoring and shows promising performance for stress inference under controlled conditions, including laboratory stress paradigms, academic tasks, online meetings, and telehealth-like settings.

However, the evidence also indicates that HRV/PRV-driven stress inference remains fragile under motion, illumination variation, short recording windows, and inconsistent reporting practices. Comparisons against wearables are informative but should be interpreted cautiously when wearable PRV/HRV is itself unstable in non-resting conditions. To move toward dependable real-world stress monitoring, future work should prioritize reference-anchored validation, standardized reporting of acquisition and preprocessing conditions, robustness testing across motion/lighting and devices, and transparent ablation-driven evaluation, especially for deep learning systems.

In summary, rPPG represents a promising but context-sensitive modality for non-contact stress and workload assessment, whose reliable deployment depends on careful validation, conservative inference strategies, and explicit acknowledgment of physiological and environmental constraints.

## Data Availability

The original contributions presented in the study are included in the article/**Supplementary Material**, further inquiries can be directed to the corresponding author.

## Supplementary material

Supplementary Table S1 Complete data extraction table for all 20 PRISMA-included studies, including study identifiers, context/protocol, acquisition parameters, rPPG pipeline, physiology/inference details, and validation information.

## References

[1] Shanghavi A Jayasuriya J Cai Y Mehta RK. Pulse rate variability from wearable wristwatches as a surrogate for heart rate variability? In: *Proceedings of the human factors and ergonomics society annual meeting (HFES)* (SAGE Publications) (2025), 1–6.

[2] Hyodo Y Kogure Y Okubo H Matsumoto N Nishiyama Y Ono T. How well can PPG be an alternative to ECG for acute stress detection in non-resting state? A comprehensive evaluation using a dnn. In: *Proceedings of the IEEE international conference on affective computing and intelligent interaction workshops (ACIIW)* (IEEE) (2023), 1–6.

[3] Charlton PH Allen J Bailón R Baker S Behar JA Chen F, et al. The 2023 wearable photoplethysmography roadmap. Physiol Meas. (2023) 44:111001. 10.1088/1361-6579/acead2 PMC10686289 37494945

[4] Zhang L Chen J Han H Lv Y Qiu S Wang C. Morphological photoplethysmography features enhance stress detection in earbud sensors. In: *Proceedings of the IEEE engineering in medicine and biology society (EMBS) international conference* (IEEE) (2024), 1–6. 10.1109/EMBC53108.2024.10782695 40039231

[5] Verkruysse W Svaasand LO Nelson JS. Remote plethysmographic imaging using ambient light. Opt Express. (2008) 16(26):21434–45. 10.1364/OE.16.021434 19104573 PMC2717852

[6] Poh M-Z McDuff DJ Picard RW. Non-contact, automated cardiac pulse measurements using video imaging and blind source separation. Opt Express. (2010) 18(10):10762–74. 10.1364/OE.18.010762 20588929

[7] Poh MZ McDuff DJ Picard RW. Advancements in non-contact, multiparameter physiological measurements using a webcam. IEEE Trans Biomed Eng. (2011) 58(1):7–11. 10.1109/TBME.2010.2086456 20952328

[8] de Haan G Jeanne V. Robust pulse rate from chrominance-based rPPG. IEEE Trans Biomed Eng. (2013) 60(10):2878–86. 10.1109/TBME.2013.2266196 23744659

[9] Wang W den Brinker AC Stuijk S de Haan G. Algorithmic principles of remote-PPG. IEEE Trans Biomed Eng. (2017) 64(7):1479–91. 10.1109/TBME.2016.2609282 28113245

[10] Chen W McDuff D. Deepphys: video-based physiological measurement using convolutional attention networks. In: *Proceedings of the European conference on computer vision (ECCV)*. (2018), 349–65.

[11] Chen X Cheng J Song R Liu Y Ward RK Wang ZJ. Video-based heart rate measurement: recent advances and future prospects. IEEE Trans Instrum Meas. (2019) 68(10):3600–15. 10.1109/TIM.2018.2879706

[12] Xiao H Liu T Sun Y Li Y Zhao S Avolio A. Remote photoplethysmography for heart rate measurement: a review. Biomed Signal Process Control. (2024) 88:105608. 10.1016/j.bspc.2023.105608

[13] Pirzada P Wilde A Harris-Birtill D. Remote photoplethysmography for heart rate and blood oxygenation measurement: a review. IEEE Sens J. (2024) 24:23436–53. 10.1109/JSEN.2024.3405414

[14] McDuff D Gontarek S Picard R. Remote measurement of cognitive stress via heart rate variability. In: *Proceedings of the IEEE engineering in medicine and biology society (EMBC)*. (2014), 2957–60. 10.1109/EMBC.2014.6944243 25570611

[15] Bousefsaf F Maaoui C Pruski A. Remote assessment of HRV to detect mental stress. In: *Proceedings of pervasivehealth*. (2013), 348–51.

[16] Frédéric B Choubeila M Alain P. Remote detection of mental workload changes using cardiac parameters assessed with a low-cost webcam. Comput Biol Med. (2014) 53:154–63. 10.1016/j.compbiomed.2014.07.014 25150821

[17] Morales-Fajardo HM Rodríguez-Arce J Gutiérrez-Cedeño A Viñas JC Reyes-Lagos JJ Abarca-Castro EA, et al. Towards a non-contact method for identifying stress using remote photoplethysmography in academic environments. Sensors. (2022) 22(10):3780. 10.3390/s22103780 35632193 PMC9146726

[18] Sun Z Vedernikov A Kykyri V-L Pohjola M Nokia M Li X. Estimating stress in online meetings by remote physiological signal and behavioral features. In: *Adjunct Proceedings of the 2022 ACM international joint conference on pervasive and ubiquitous computing and the 2022 ACM international symposium on wearable computers*. UbiComp/ISWC ’22 Adjunct. (New York, NY, USA: Association for Computing Machinery) (2023), 216–20. 10.1145/3544793.3563406

[19] Moustafa I Soteafy S Hassanein H. Remote livestream-based stress assessment in telehealth services. In: *Proceedings of the IEEE Canadian conference on electrical and computer engineering (CCECE)* (IEEE) (2024), 1–6.

[20] Chen F Kong L Zhao Y Dong L Liu M Hui M. Non-contact measurement of mental stress via heart rate variability. In: Tescher AG, Ebrahimi T, editors. *Applications of Digital Image Processing XLIII*. Bellingham, WA: SPIE (2020). Vol. 11510. p. 306–13. 10.1117/12.2567245

[21] Nikolaiev S Telenyk S Tymoshenko Y. Non-contact video-based remote photoplethysmography for human stress detection. J Autom Mob Rob Intell Syst. (2019) 14:63–73. 10.14313/JAMRIS/2-2020/21

[22] Iuchi K Mitsuhashi R Goto T Matsubara A Hirayama T Hashizume H, et al. Stress levels estimation from facial video based on non-contact measurement of pulse wave. Artif Life Rob. (2020) 25(3):335–42. 10.1007/s10015-020-00624-4

[23] McDuff D Nishidate I Nakano K Haneishi H Aoki Y Tanabe C, et al. Non-contact imaging of peripheral hemodynamics during cognitive and psychological stressors. Sci Rep. (2020) 10:10884. 10.1038/s41598-020-67647-6 32616832 PMC7331808

[24] Finotti G Di Lernia D Tsakiris M Riva G Naber M. Remote photoplethysmography (rPPG) in the wild: remote heart rate imaging via online webcams. Behav Res Methods. (2024) 56:6904–14. 10.3758/s13428-024-02398-0 38632165 PMC11362249

[25] Khomidov M Lee D Kim C-H Lee J-H. The real-time image-sequences-based stress assessment vision system for mental health. Electronics. (2024) 13(11):2180. 10.3390/electronics13112180

[26] Fontes L Machado P Vinkemeier D Yahaya S Bird JJ Ihianle IK. Enhancing stress detection: a comprehensive approach through rppg analysis and deep learning techniques. Sensors. (2024) 24(4):1096. 10.3390/s24041096 38400254 PMC10892284

[27] Xu J Song C Yue Z Ding S. Facial video-based non-contact stress recognition utilizing multi-task learning with peak attention. IEEE J Biomed Health Inform. (2024) 28(9):5335–46. 10.1109/JBHI.2024.3412103 38861440

[28] Ziaratnia S Laohakangvalvit T Sugaya M Sripian P. Multimodal deep learning for remote stress estimation using CCT-LSTM. In: *Proceedings of the IEEE winter conference on applications of computer vision (WACV)* (IEEE) (2024), 1–10.

[29] Hendryani A Rizkinia M Gunawan D. Enhancement of stress classification using web camera-based imaging photoplethysmography with a frame alignment method. IEEE Access. (2024) 12:122313–27. 10.1109/ACCESS.2024.3452934

[30] Du Y Yang H. Remote assessment of physiological parameters by non-contact methods to detect mental stress. In: *International conference on image, signal processing, and pattern recognition (ISPP 2025)* (SPIE) (2025), 13664, 136643M.

[31] Liu I Liu F Zhong Q Ma F Ni S. Your blush gives you away: detecting hidden mental states with remote photoplethysmography and thermal imaging. PeerJ Comput Sci. (2024) 10:e1912. 10.7717/peerj-cs.1912 38660202 PMC11041963

[32] Ouzar Y Lagha L Bousefsaf F Maaoui C. Multimodal stress state detection from facial videos using physiological signals and facial features. In: *Proceedings of the international conference on affective computing and intelligent interaction* (Springer) (2023), 1–12.

[33] Lampier LC Caldeira E Delisle-Rodriguez D Floriano A Bastos-Filho TF. A preliminary approach to identify arousal and valence using remote photoplethysmography from face video. In: *XXVII Brazilian congress on biomedical engineering (IFMBE proceedings)*. (2022), 83, 1659–64.

[34] van Es VAA Lopata RGP Scilingo EP Nardelli M. Contactless cardiovascular assessment by imaging photoplethysmography: a comparison with wearable monitoring. Sensors. (2023) 23(3):1505. 10.3390/s23031505 36772543 PMC9919512

[35] Yu S-G Kim S-E Kim NH Suh KH Lee EC. Pulse rate variability analysis using remote photoplethysmography signals. Sensors. (2021) 21(18):6241. 10.3390/s21186241 34577448 PMC8471146

[36] Finžgar M Podržaj P. Feasibility of assessing ultra-short-term pulse rate variability from video recordings. PeerJ. (2020) 8:e8342. 10.7717/peerj.8342 31938579 PMC6953345

[37] Takeuchi H Ohsuga M Kamakura Y. A study on region of interest in remote ppg and an attempt to eliminate false positive results using SVM classification. In: *2021 IEEE international conference on artificial intelligence in engineering and technology (IICAIET)*. (2021), 1–5.

[38] Beh W-K Wu Y-H Wu A-Y. Robust PPG-based mental workload assessment system using wearable devices. IEEE J Biomed Health Inform. (2023) 27(5):2323–33. 10.1109/JBHI.2021.3138639 34962889

[39] Li S Elgendi M Menon C. Optimal facial regions for remote heart rate measurement during physical and cognitive activities. npj Cardiovasc Health. (2024) 1:3. 10.1038/s44325-024-00033-7 41775888 PMC12912399

[40] Bobbia S Macwan R Benezeth Y Mansouri A Dubois J. Unsupervised skin tissue segmentation for remote photoplethysmography. Pattern Recognit Lett. (2019) 124:82–90. 10.1016/j.patrec.2017.10.017

[41] Berntson GG Thomas Bigger Jr. J Eckberg DL Grossman P Kaufmann PG Malik M, et al. Heart rate variability: origins, methods, and interpretive caveats. Psychophysiology. (1997) 34(6):623–48. 10.1111/j.1469-8986.1997.tb02140.x 9401419

[42] Kim H-G Cheon E-J Bai D-S Lee YH Koo B-H. Stress and heart rate variability: a meta-analysis and review of the literature. Psychiatry Investig. (2018) 15(3):235–45. 10.30773/pi.2017.08.17 29486547 PMC5900369

[43] Billman GE. The LF/HF ratio does not accurately measure cardiac sympatho-vagal balance. Front Physiol. (2013) 4:26. 10.3389/fphys.2013.00026 23431279 PMC3576706

[44] Lee S Song YD Lee EC. Ultra-short-term stress measurement using RGB camera-based remote photoplethysmography with reduced effects of individual differences in heart rate. Med Biol Eng Comput. (2025) 63(2):497–510. 10.1007/s11517-024-03213-w 39392540

[45] Meziati Sabour AA Guyard P-A Fauchon GL Aouayeb N Pezel A Boulanger PLH, Ubfc-phys: a multimodal database for psychophysiological studies of social stress. IEEE Trans Affect Comput. (2023) 14(1):622–36. 10.1109/TAFFC.2021.3056960

[46] Heusch G Marcel S Anjos A. Cohface dataset (2016).

[47] Niu X Han H Shan S Chen X. VIPL-HR: a multi-modal database for pulse estimation from less-constrained face video. In: *Asian Conference on Computer Vision (ACCV)*. (2018).

[48] Tang J Chen K Wang Y Shi Y Patel SN McDuff D, et al. MMPD: multi-domain mobile video physiology dataset. In: *45th annual international conference of the IEEE engineering in medicine & biology society (EMBC)*. (2023), 1–5. 10.1109/EMBC40787.2023.10340857 38083085

[49] Rodrigues G Garcia NM. Evaluating remote photoplethysmography: a 10-minute video dataset in uncontrolled lighting. Data Brief. (2025) 61:111888. 10.1016/j.dib.2025.111888 40747439 PMC12311941

[50] Koldijk S Sappelli M Verberne S Neerincx MA Kraaij W. The swell knowledge work dataset for stress and user modeling research. In: *Proceedings of the 16th international conference on multimodal interaction (ICMI)* (ACM) (2014), 291–8.

[51] Yu X Paul M Antink CH Venema B Blazek V Bollheimer C, et al. Non-contact remote measurement of heart rate variability using near-infrared photoplethysmography imaging. In: *2018 40th Annual international conference of the IEEE engineering in medicine and biology society (EMBC)*. (2018). p. 846–9. 10.1109/EMBC.2018.8512451 30440524

[52] Odinaev I Wong KL Chin JW Goyal R Chan TT So RHY. Robust heart rate variability measurement from facial videos. Bioengineering. (2023) 10(7):851. 10.3390/bioengineering10070851 37508878 PMC10376629

[53] Castellano Ontiveros M Elgendi M Menon C. A machine learning-based approach for constructing remote photoplethysmogram signals from video cameras. Commun Med. (2024) 4:109. 10.1038/s43856-024-00519-6 38849495 PMC11161609

[54] van der Kooij KM Naber M. An open-source remote heart rate imaging method with practical apparatus and algorithms. Behav Res Methods. (2019) 51:2106–19. 10.3758/s13428-019-01256-8 31152386 PMC6797647

[55] Liu X Narayanswamy G Paruchuri A Zhang X Tang J Zhang Y, et al. rPPG-toolbox: deep remote PPG toolbox. In: *Advances in neural information processing systems (NeurIPS), datasets and benchmarks track*. (2023).

[56] Heusch G Anjos A Marcel S. A reproducible study on remote heart rate measurement. (2016).

[57] Odinaev I Prae-arporn K Wong KL Chin JW Chan TT Goyal R. Camera-based heart rate variability and stress measurement from facial videos. In: *Proceedings of the IEEE/ACM conference on connected health: applications, systems and engineering technologies (CHASE)*. (2022).

[58] Mortensen JA Mollov ME Chatterjee A Ghose D Li FY. Multi-class stress detection through heart rate variability: a deep neural network based study. IEEE Access. (2023) 11:57470–80. 10.1109/ACCESS.2023.3274478

[59] Dalmeida KM Masala GL. HRV features as viable physiological markers for stress detection using wearable devices. Sensors. (2021) 21(8):2873. 10.3390/s21082873 33921884 PMC8072791

[60] Elgendi M Martinelli I Menon C. Optimal signal quality index for remote photoplethysmogram sensing. npj Biosensing. (2024) 1:5. 10.1038/s44328-024-00002-1

[61] Dasari A Prakash SKA Jeni LA Tucker CS. Evaluation of biases in remote photoplethysmography methods. npj Digit Med. (2021) 4:91. 10.1038/s41746-021-00462-z 34083724 PMC8175478

[62] Nowara E McDuff D Veeraraghavan A. A meta-analysis of the impact of skin type and gender on non-contact photoplethysmography measurements. In: *IEEE/CVF conference on computer vision and pattern recognition workshops (CVPRW)*. (2020).

[63] Bondarenko M Menon C Elgendi M. Demographic bias in public remote photoplethysmography datasets. npj Digit Med. (2025) 8:593. 10.1038/s41746-025-01973-9 41038972 PMC12491395

